# African researchers do not think differently about Open Data

**DOI:** 10.3389/frma.2022.950212

**Published:** 2022-07-15

**Authors:** Lara Skelly, Elisha R. T. Chiware

**Affiliations:** ^1^Loughborough University Library, Loughborough University, Loughborough, United Kingdom; ^2^Stellenbosch Business School, Stellenbosch University, Stellenbosch, South Africa; ^3^Cape Peninsula University of Technology Library, Cape Peninsula University of Technology, Cape Town, South Africa

**Keywords:** Open Data, Africa, Open Science, attitudes, longitudinal

## Abstract

A key motivation for Open Science is accessibility. For researchers in resource-poor economies, this translates into access to the methods, data and publications that will foster scientific research and discovery in such communities and environments. Attitudes toward Open Science are in flux, and there is a growing awareness of the roles and responsibilities that researchers have to one another in this regard. This paper explores how African researchers approach issues relating to Open Data by reporting on the *State of Open Data Report* data. Focusing on the attitudes toward Open Data, this paper reports on how African researchers view (i) data sharing, (ii) the use of shared data, and (iii) the Open Data ecosystem. The findings show that, although the attitudes of African researchers have changed over time, they are not very different from those held by their international counterparts. These findings will aid policymakers, as well as academic and research institutions, in highlighting the areas of future growth for Open Data in Africa.

## Introduction

Scientific research in the 21st century has been scaled to new practices where scientists work more collaboratively and in data-intensive environments (Tenopir et al., 2011). Advances in technology have enabled this increased scale, as well as the consistently high levels of investments and development in research infrastructures. The emergence of Open Science principles and the insistence by governments and leading global research funders to make publicly funded research more open and accessible for the public good is advancing data sharing in and across research domains. This collaborative and multidisciplinary nature of scientific research leads to significant changes in how research is conducted and, more importantly, how research data is managed and preserved. New ‘best practice’ procedures and resources are being enabled by the adoption of the Findable, Accessible, Interoperable and Reusable (FAIR) practices, which include data accessibility, discovery, reuse, preservation and, particularly, data sharing (Wilkinson et al., 2016). The other major driver of global Open Data sharing practices among scientific communities is the realization of the importance of community involvement through citizen science, especially in data collection and the utilization of research outputs in those communities.

The Royal Society (2012) points out that the “publication of scientific theories and the experimental and observational data on which they are based, permits others to identify errors, to support, reject or refine theories and to reuse data for further understanding and knowledge”. Bird and Frey (2013) also emphasize that “Open Science entails the sharing of more than mere fact”. A fundamental benefit of openly sharing data is safeguarding resources, especially time and money, while gaining knowledge leverage. Without systematic data processing that assigns the attribution of intellectual property to those who created data, scientists will always be skeptical about openly sharing data. The lack of clarity around attribution, quality, and responsibility erodes trust in sharing and complicates processes. In addition, data, due to its complexity and variability, if not properly managed, may not be discovered.

Consequently, research data management is crucial in making data systematically and logically storable, discoverable and accessible, and curated with appropriate metadata. Funding agencies, national governments and entities supporting research are enforcing a range of requirements to enable the long-term preservation and sharing of research data by encouraging both research publications and results that are “open” and readily accessible for the benefit of humankind and knowledge development. Ramsay (2022) emphasizes the important point that “data sharing is essential to the advancement of science”, and Tenopir et al. (2011) also state that data sharing is a valuable part of the scientific method allowing for verification of results and extending research from prior results.

Various scientific research disciplines have, over the years, developed their own systems and protocols for sharing data both in and out of the laboratory. There has never been a mandatory approach to how data should be shared. Over the last decade and going into the future, the scientific community is witnessing a deluge of data and, consequentially, the development of systems to support the management of large data sets. In Chemistry, researchers are said to be lagging in recognizing the importance and value of curating their data and information for the purposes of exchanging it (Bird and Frey, 2013). Bird and Frey (2013) further point out that “the growth and complexity of datasets produced have encouraged the expansion of e-Research, and stimulated the development of methodologies for managing, organizing, and analyzing ‘big data”’. The growing e-Science and e-Research practices now underpin scientific research projects. Furthermore, calls from research funders for open access practices to research outputs and, more importantly, Open Data management approaches are driving research disciplines to find new solutions to share data.

In 2011, Tenopir et al. (2011) showed that in various researchers' practices, “barriers to effective data sharing and preservation were deeply rooted in the practices and culture of the research process as well as the researchers themselves”. By 2015, Tenopir et al. (2015), continuing on the same research that tracks researchers' data sharing practices, reported a shift in behavior. Their results point to (i) the increased acceptance of and willingness to engage in data sharing and (ii) an increase in actual data-sharing behaviors. Tenopir et al. (2015) further noted increased perceptions about the risk associated with data sharing and that specific barriers to data sharing persisted. It was also reported that there are differences across age groups, with younger respondents feeling more favorably toward data sharing and reuse yet making less of their data and research available or “open” than older respondents. The generational differences could be attributed to fear of competition in career development and promotion. Geographic differences were also noted to exist and were understood in terms of collectivist and individualist cultural differences.

Through the *State of Open Data Reports*, the levels of data sharing and usage have been monitored since 2016 in over 190 countries. The *2021 Report* goes beyond the usual metrics and includes new topics on “What motivates researchers to share data and the perceived discoverability and credibility of data shared openly”. The key findings of this report are that (i) there is more concern about sharing datasets than ever before, (ii) there is more familiarity and compliance with the FAIR data principles than ever before, and (iii) repositories, publishers, and institutional libraries have a pivotal role to play in helping make data openly available.

In Africa, several researchers have explored the data sharing practices among researchers and have identified a number of barriers and opportunities. These barriers to data sharing in African research institutions are mainly associated with (i) the lack of policy and guideline frameworks at institutional and national levels, (ii) limited funding and (iii) inadequate infrastructures (Bezuidenhout, 2017; Bezuidenhout and Chakauya, 2018; Bangani and Moyo, 2019; Chiware, 2020; Abebe et al., 2021). The research in Africa and other developing and low-income environments on data sharing, has mainly focused on data sharing in public health and medical research.

Therefore, the purpose of this paper was to explore whether the attitudes of African researchers are different from those in other environments concerning data sharing and its practices. The paper analyzed data from *The State of Open Data Reports* (Hyndman, 2018) to answer these questions. More specifically, the analysis was centered around researchers' attitudes toward the following three areas: (i) the sharing of their own data, (ii) the shared data of others, and (iii) the Open Data ecosystem in place to enable wider data sharing. It was found that the attitudes of African researchers have changed over time, but they are not very different from their international counterparts.

## Literature review

Africa provides a complex context within which the topic of Open Data has been explored and written up in the literature. This context can be viewed from an individual, policy, or resource perspective. These contexts inform the researchers' attitudes toward sharing their own data, using shared data, and the wider Open Data ecosystem.

From the individual context perspective, an exploration of South African and Kenyan biochemistry researchers' data sharing perspectives revealed that individual perceptions of research environments were highly influential in shaping data sharing practices. Current low perceptions could be addressed through discussions on incentives and approaches that will improve the overall weak research environment (Bezuidenhout, 2017). In a study entitled “Narratives and counternarratives on Data Sharing in Africa”, Abebe et al. (2021) argue that the many narratives emerging on data sharing among African scientists are somewhat distorting the full complexity of the African data sharing landscape where obstacles, issues, and challenges of data sharing on the continent are multifaceted. Anane-Sarpong et al. (2020) also believe that much more empirical research that engages stakeholders in data sharing in African research is still required to further and better understand the multitude of Open Science and Open Data challenges.

Understandably, there are serious concerns due to unclear institutional and national policy frameworks that currently guide research data sharing in many African countries, where the approaches are said to be in a piecemeal fashion and are further complicated by an uneven global *Open Data* sharing framework that is dictated by national and regional interests (Bezuidenhout, 2017). Stein (2020) points out that data sharing is an important aspect of science and is now required by most funding bodies and journals. Abebe et al. (2021) see entrenched and unbalanced historical power dynamics, trust issues, the need to better understand the African context, and the disregard of African generated research at play.

The national regulatory guidelines of many African nations do not explicitly allow for broad genetic data sharing, for example, and there is a need to reconsider these policies and propose creative solutions. In African genomic data sharing, Ramsay (2022) argues that despite a steady increase in data in international repositories, very little is coming from Africa and that the current analysis of genome data from Africa has yielded over 3 million novel and previously undocumented variants that could benefit the global community. Anane-Sarpong et al. (2020) also established that in the broader health sector, the impediments to data sharing include, among others, (i) risks faced by under-resourced researchers and their institutions, which have no capacity to quickly generate data produced into new knowledge, (ii) the lack of integrated guidelines and support mechanisms to address risk and reward researchers, and (iii) the general lack of confidence in existing protective safeguards.

From a resource perspective, Alter and Vardigan (2015) established that the many barriers to data sharing among researchers in low and middle-income countries are around (i) informed consent, (ii) data management, (iii) data dissemination, and (iv) the validation of research contributions. Ramsay (2022) also points to the challenges in the research ecosystems, including brain drain, lack of opportunities for young researchers, and limited resources. Denny et al. (2015) suggest that “for data sharing to be effective and sustainable, multiple social and ethical requirements need to be met and that an effective model of data sharing will be one in which considered judgments will need to be made about how best to achieve scientific progress, minimize risks of harm, promote fairness and reciprocity, and build and sustain trust”.

The growth and development of data repositories are still limited in African institutions. However, those receiving international funding and who are publishing in international journals might be facing new requirements that ultimately require them to openly share data through different disciplinary and publishers' platforms. Bezuidenhout and Chakauya (2018) further elaborate on the “hidden concerns of sharing research data by low/middle-income country scientists” by pointing to the uneven landscape in which these scientists are equated when compared to their counterparts in developed environments that have far more developed and stable infrastructures. Despite these barriers, there is a growing interest among lower and middle-income scientists in data sharing (Bezuidenhout and Chakauya, 2018).

These contexts inform researchers' attitudes. Recent global surveys show that attitudes toward data sharing, data use, and data reuse are primarily positive, and that behavior does not always support these attitudes. Assistance through (i) Data Managers or Data Librarians, (ii) readily available data repositories for both long-term and short-term storage, and (iii) educational programs for both awareness and to help engender good data practices are needed (Tenopir et al., 2020). Bangani and Moyo (2019) observe that in South African universities, researchers preferred to use data produced by others and were not open to sharing their own data.

A study by Thoegersen and Borlund (2021) on researcher attitudes toward data sharing in public data repositories shows a need for greater clarity and consistency in using the term “*data sharing”* in future studies to better understand the use of this term, its phenomenon and to allow for cross-study comparison. In reviewing social scientists' data-sharing behaviors, Kim and Adler (2015) established that personal motivations and norms of data-sharing supported data sharing practices. However, institutional pressures by funding agencies, journals and data repositories required encouragement to facilitate social scientist data-sharing behaviors.

## Methods

This article uses data collected for *The State of Open Data Report* in the 2017 and 2021 waves. The study is a longitudinal study, funded by the International Research Center, with the support of the Open Data for Development (OD4D) Network, and conducted by Digital Science, SpringerNature and Figshare. The data for the various waves, including the associated questionnaires and reports, are freely available (Hyndman, 2018).

The survey covers various topics relating to Open Data, including attitudes and experiences of researchers toward data sharing across multiple research domains. Relevant to this article are the attitudes of the African researchers. Most questions are closed, categorical-type questions, limiting the statistical tests that could be performed. To conduct a comparative analysis, simple percentages were used and evaluated.

The sample is collated on the question, “Which country/territory are you located in” and, given the international fluidity of researchers, it is conceivable that those responding that they are located currently in Africa would not necessarily all originate from Africa. Among those who originate from Africa and are presently still in Africa, these researchers would likely have had international exposure through travel or collaboration with others. However, for ease of reference, the respondents who gave “Africa” as their continent of location will be termed “African researcher”.

The first year that the *State of Open Data Report* was prepared was 2016. In that year, no responses were received from the African continent. The following year, 2017, saw 151 responses. Some of the questions asked in that year were repeated in 2021, providing a longitudinal insight over 5 years.

This study on whether African researchers view Open Data sharing differently from their non-African counterparts was framed within a three-layered framework: (i) attitudes to sharing one's own data, (ii) attitudes to the use of shared data, and (iii) attitudes to the broader sharing ecosystem. The framework can also be viewed within the overall research landscape in Africa as to whether the correct policies, incentives and infrastructures exist to enable positive attitudes toward the advancement of science on the continent and globally. The study on attitudes toward data sharing in Africa can also be viewed in the context of the conclusions from Baždarić et al. (2021) that this has to be framed within the broader understanding and appreciation of the principles and practices of Open Science.

Kim and Adler (2015) explored data-sharing behaviors on individual motivations, institutional pressures, and pressures using a combination of new institutional theory and the theory of planned behavior to develop a model that explains and predicts data sharing behavior. The suggested framework for this study is closely related to the new institutional theory and the theory of planned behavior. The exploration was on whether African researchers think differently about Open Data considering their personal positions and the conditions within their institutions.

## Results

Research on data sharing practices globally and in Africa has been ongoing. Several outputs already provide details on how new approaches are being adopted or shunned globally. It is important to note what Abebe et al. (2021) terms narratives and counternarratives on African data sharing. To a large extent, recent literature on the subject has tended to ignore the African reality that is driven by (i) the lack of both developed research infrastructures and (ii) coordinated institutional and national policy frameworks on data sharing and the broader Open Science environments.

Given this wealth of information that is already available on the attitudes of African researchers toward Open Data, this article contributes by using new data to either confirm or refute existing findings.

### Data comparison of attitudes between africa and the rest of the world

#### Attitudes to sharing one's own data

African researchers are no different from their counterparts in other regions when comparing their reported comfort levels relating to how other researchers might reuse their data. Replication, reanalysis, reinterpretation, isolated reuse, and combination reuse are globally accepted as expected forms of reusing data.

African researchers share much of the same challenges that other researchers experience. The uncertainty of sharing rights, ethical considerations and other permissions are of universal concern. African researchers are less concerned with the lack of time, size of datasets, organizing data and the risk of being 'scooped', while they are more unsure about which repository to use.

Motivations to share data are no different on the African continent than elsewhere. Public benefit and increased impact are given as strong circumstances that would motivate the sharing of data, which speaks to the altruistic attitudes of researchers. Additional key motivators are the recognitions, whether through full data citation, citation of research papers or co-authorships. Non-African researchers report considerably more frequently that the citation of research papers is a possible motivator (61% of non-African researchers, while only 29% of African researchers). Non-African researchers give the citation of research papers as the primary motivator for sharing data, while African researchers reflect that public benefit is what motivates them.

#### Attitudes to using Open Data

Researchers throughout the surveyed countries reported that shared data offers benefits: it (i) fosters collaboration, (ii) validates findings, (iii) complements existing data and (iv) avoids the duplication of efforts. Whether these benefits are experienced, or just a perception of potential benefits is unclear. Regardless, the survey participants indicated that those who share their data do not receive sufficient recognition for their data. If recognition is a primary motivator, as reported in the previous section, then policies that follow those climates that foster recognition would also increase the sharing of research data.

A substantial majority of the respondents (79%) indicated that shared data added to the credibility of the research. As public benefit is a strong motivator, spreading the perception of shared data that is linked to research credibility would likely increase data sharing practices.

When participants were asked how they determined the quality of the shared data, many factors were considered and shown to be relevant, including (i) the reputation of the source of the data, (ii) the associated peer-reviewed article, and (iii) the availability of visualizations that are consistent with the data. Non-African researchers reported that clear dataset descriptions, which provide sufficient context, are a strong indicator of quality (84%), an opinion that was not as universally held by the African respondents: only 41% of them reported this to be the case. All researchers agreed that datasets that are easy to find are more likely to be viewed as credible.

#### Attitudes to the Open Data ecosystem

There is global agreement reported from the study participants that national mandates to make research data openly available to access, reuse, repurpose and redistribute would be welcomed since a mere 7% of the respondents disagreed. There is also support for funders to mandate data sharing as part of their grant awards, with 53% being in favor and 27% being against such a policy.

When asked whether making research articles open access should be common scholarly practice, the view was strongly affirmative: 93% of the African respondents and 87% of the non-African respondents agreed. The adoption of Open Data practices appears to have slightly less traction where 84% of the African respondents and 80% of the non-African respondents agreed.

### Data comparison of African attitudes over time

The participants who responded in 2017 were from varied countries, with most responses originating from the more research-active countries: Egypt, Nigeria and South Africa. More than 60% came from universities, a distribution that is essentially unchanged in 2021.

#### Attitudes to sharing one's own data

In the 2017 questionnaire, fewer options were given to respondents in answer to the question of *what would motivate them*. Only a quarter of the respondents gave *being cited* as a motivator, while the *ease of sharing data* and *freedom of information request* was much stronger. It would seem that African researchers have become more aware of the factors motivating the sharing of data in the past 5 years, which speaks to a greater awareness of the Open Science movement. As in the 2021 data, respondents of the 2017 questionnaire gave *public benefit* as a strong motivator. Furthermore, *co-authorship credit* as a motivator has increased considerably over the past 5 years.

#### Attitudes to using Open Data

Only one question relating to the attitudes toward using Open Data was asked in 2017: *How do you think the data shared by others have or could benefit you?* The data comparison between the 2017 and 2021 responses shows that African researchers appear to have a greater awareness of the personal benefits of Open Data. The ambiguous phrasing of the question makes it unclear if the attitudes to the benefits resulted from a personal experience or simply a reflection of the current narrative among researchers.

#### Attitudes to the Open Data ecosystem

Support for a national mandate has increased considerably. In 2017, 33% of the respondents took a neutral position, and 54% supported the statement relating to national mandates for Open Data. In 2021, those taking the neutral position had dropped to only 6%, with an overwhelming 87% supporting a national mandate.

As the number of respondents doubled over that time, one can surmise that there has been an increased interest in issues relating to Open Data in the past 5 years. This increased awareness and interest is reflected in the other questions asked.

## Discussion

This study focused on whether African researchers think differently about Open Data compared with their global peers, and whether the thinking of African researchers have changed over time. The findings reported here illustrate that, in general, research stakeholders are supportive of data sharing, with African researchers' practices and experiences not very different from their international counterparts, despite the policy differences. It is clearly highlighted that African researchers' attitudes toward data sharing have changed positively over the past decade. It is affirming to know that the impact that the global Open Science movement's message on the benefits of data sharing is spreading across the globe and to the different research communities. The changing positive attitudes by African researchers toward data sharing could be attributed to the new requirements by journal publishers and research funders that data outputs must be visible to knowledge consumers.

The results further indicate that by 2021 African researchers were more supportive of their national governments' mandates to share data within and across research domains. There are growing efforts across the continent to formulate Open Science policy frameworks both at national and institutional levels. The United Nations Educational and Scientific Organisation has the *UNESCO Recommendation on Open Science*, a framework for member nations to develop their Open Science policy frameworks. Other donors and regional organizations like the Electronic Information for Libraries and the West and Central African Research and Education Network have also supported the development of national mandates that can assist researchers in participating in data sharing activities. In Southern Africa, especially South Africa, the Open Science environment has developed rapidly. South Africa has also moved a step further through a partnership with the European Union's *Europe Open Science Cloud* to develop its own South African Open Science Cloud framework, further supporting the about-to-be finalized South African Open Science Framework.

This study also highlights several issues that require attention from African academic and research institutions to support researchers' data sharing practices that will enable best science advancement and societal engagement. There should be clear national and institutional policy frameworks to enable good data sharing practices to take root among African researchers. The position of international research funders, journal publishers, and inter-institutional and country collaborations need to be spelt out in future policies to ensure equitable data custodianship in African generated research. Given the attitudes of African researchers toward such policies, the time is right to put them into place.

The African academic and research communities should not be left out and lose focus of the potential benefits of Open Data for reproducibility and efficiency in research, especially in poorly resourced environments that require more collaborative use of infrastructure and resources. The potential gains for further and faster benefits in advancing science and knowledge production are all too evident in coordinated data sharing activities.

## Data availability statement

Publicly available datasets were analyzed in this study. This data can be found here: Figshare repository https://doi.org/10.6084/m9.figshare.c.4046897.v6.

## Author contributions

LS took the lead in the conceptualization, methods, results, and final editing. EC took the lead in introduction, literature review, and conclusion. Both authors contributed to the article and approved the submitted version.

## Funding

This work has been partially funded by Stellenbosch Business School.

## Conflict of interest

The authors declare that the research was conducted in the absence of any commercial or financial relationships that could be construed as a potential conflict of interest.

## Publisher's note

All claims expressed in this article are solely those of the authors and do not necessarily represent those of their affiliated organizations, or those of the publisher, the editors and the reviewers. Any product that may be evaluated in this article, or claim that may be made by its manufacturer, is not guaranteed or endorsed by the publisher.
